# Fast-Convergence Reinforcement Learning for Routing in LEO Satellite Networks

**DOI:** 10.3390/s23115180

**Published:** 2023-05-29

**Authors:** Zhaolong Ding, Huijie Liu, Feng Tian, Zijian Yang, Nan Wang

**Affiliations:** 1 School of Information Science and Technology, ShanghaiTech University, Shanghai 201210, China; 2 Innovation Academy for Microsatellites of CAS, Shanghai 201304, China

**Keywords:** LEO satellite networks, satellite routing, multi-agent reinforcement learning, distributed routing

## Abstract

Fast convergence routing is a critical issue for Low Earth Orbit (LEO) constellation networks because these networks have dynamic topology changes, and transmission requirements can vary over time. However, most of the previous research has focused on the Open Shortest Path First (OSPF) routing algorithm, which is not well-suited to handle the frequent changes in the link state of the LEO satellite network. In this regard, we propose a Fast-Convergence Reinforcement Learning Satellite Routing Algorithm (FRL–SR) for LEO satellite networks, where the satellite can quickly obtain the network link status and adjust its routing strategy accordingly. In FRL–SR, each satellite node is considered an agent, and the agent selects the appropriate port for packet forwarding based on its routing policy. Whenever the satellite network state changes, the agent sends “hello” packets to the neighboring nodes to update their routing policy. Compared to traditional reinforcement learning algorithms, FRL–SR can perceive network information faster and converge faster. Additionally, FRL–SR can mask the dynamics of the satellite network topology and adaptively adjust the forwarding strategy based on the link state. The experimental results demonstrate that the proposed FRL–SR algorithm outperforms the Dijkstra algorithm in the performance of average delay, packet arriving ratio, and network load balance.

## 1. Introduction

Terrestrial communications already meet much of our daily communication needs, but for users in remote areas and at sea, their communications needs cannot be met. Unlike terrestrial communication, satellite communication has a longer range and better communication quality [1]. It meets the communication needs of users in remote areas and at sea, and can also be used to complement terrestrial communications to serve urban users. Therefore, satellite communication research has attracted much attention.

Based on the distance of the orbital plane from the Earth’s surface, satellite systems can be classified as geostationary Earth orbit (GEO), medium Earth orbit (MEO), and low Earth orbit (LEO) satellite systems. Unlike GEO and MEO, LEO satellites are usually located in the orbital plane, at an altitude of 500 to 2000 km [2]. Because of low latency, low path loss, and low launch costs, LEO satellite networks are a major research direction for the industry. At present, there are many large LEO satellite constellations in operation, such as Starlink and Oneweb. The link status of the satellite network LEO is volatile due to the complex space environment and frequent satellite laser failures. In addition, satellites move at high speeds, resulting in frequent changes in satellite topology. These two characteristics are the main differences between satellite networks and terrestrial networks. Therefore, traditional routing algorithms are not suitable for direct use for satellite networks. New routing algorithms need to be designed to solve the LEO satellite routing problem.

Typically, the routing problem of the LEO satellite is mainly divided into two parts: how to obtain the network topology, and how to generate the route paths based on the network topology [3]. Mauger [4] first proposed the concept of virtual node. When the satellite moves to the virtual node location, its logical address becomes the logical address of that virtual node. The routing method is to give priority to the path with the highest latitude. The authors in [5] proposed the dynamic virtual topology routing (DV–DVTR), which divided the system period into time slices. For each time slot, the network topology was considered to be fixed [6], and then packets were forwarded according to the shortest path first algorithm. Although DV–DVTR is easy to implement, the division of time intervals is a very difficult task. Smaller time intervals require more storage space, and larger time intervals affect the performance of the algorithm. The authors in [7] proposed the Temporal Netgrid Model (TNM) to portray the time-varying topology of LEO satellite networks, in which the Dijkstra algorithm was used to generate forwarding policies. The Dijkstra algorithm needs to obtain global network information to operate, which can cause an increase in the communication load on the satellite network. Also, the link state of the satellite network changes very quickly and, by the time the node has collected the global information, much of it may be invalid. The authors in [8] proposed a distributed routing method, in which the surface was divided into several spaces with corresponding logical areas. In [9], each node sent congestion information to neighbor nodes, including queue length and available resources. Therefore, satellite nodes could route packages based on congestion information to achieve load balancing.

There has been a significant amount of research on the application of Software Defined Network (SDN) technology to address satellite routing challenges. For example, a study conducted in [10] investigated a LEO satellite routing algorithm in a software-defined space terrestrial integrated network, where SDN technology was applied to the LEO satellite network. The lattice-like topology of the satellite network created a shared bottleneck problem, which was addressed in the study. To tackle these issues, ref. [11] proposed an SDN-coordinated Multipath TCP (scMPTCP) architecture and related algorithms. In [12], the authors aimed to introduce an SDN-based terrestrial satellite network architecture and estimate the mean time required to transport data of a new traffic flow from the source to the destination while considering the time required to transfer SDN control actions. It should be noted that the proposed algorithms in these studies were centralized routing algorithms, which require frequent communication with the ground station and may cause some latency, presenting a significant disadvantage.

The above approaches mainly consider how to shield network dynamics and then run traditional routing algorithms for static network topology. These methods take up a certain amount of storage space and do not yield accurate satellite network topology. In recent years, a lot of research has started to use machine learning methods to solve routing problems. The difference between machine learning methods and traditional methods is that the former are data-driven, while the latter are model-driven. If the model does not describe the problem accurately, the performance of the model-driven method will be poor. Recently, machine learning has been applied in network areas, such as regulating congestion at the transport layer [13], optimizing video stream transmission [14], allocating resources [15], and so on.

The most suitable machine learning method for solving routing problems is the reinforcement learning technique. In [16], they used the Q-routing method to decide how to forward packages in the LEO satellite network. In [17], deep reinforcement learning was used to solve the routing problem; they used the neural network to replace Q-tables to store Q values. They both use centralized routing algorithms that viewed all satellite nodes as the agent that learned packet forwarding policies as it interacted with the network. The disadvantages of this approach are the need to collect global link state information and the high signaling overhead. The authors in [18] proposed a dynamic distributed routing scheme based on reinforcement learning; they considered each satellite node as an agent, and agents trained and executed routing operations. However, this did not consider the problem of the convergence speed of the algorithm, but simply provided the approximate running process of the algorithm.

Even though many researchers have applied reinforcement learning to routing problems [19], few of them have improved the convergence speed of the algorithm to face the dynamically changing link state. In this paper, we propose a distributed reinforcement learning method named FRL–SR, which not only learns routing and forwarding policies by distributed reinforcement learning, but also accelerates the convergence speed of the algorithm and senses the satellite network state faster. Our main contributions can be summarized as follows:

- We propose a distributed reinforcement learning method named FRL–SR; it has lower end-to-end latency and lower signaling overhead than traditional algorithms;
- We propose a learning mechanism that allows the algorithm to converge faster in the face of changes in the network link state;
- We compare the impact of different reward functions on the performance of reinforcement learning algorithms. The experimental results show that the design of reward functions is crucial for reinforcement learning algorithms.

The remainder of this paper is organized as follows. In Section 2, we describe the system model and Q-routing algorithm. In Section 3, we give the details of our FRL–SR method. We discuss the experimental results in Section 4. Finally, Section 5 concludes our work.

## 2. System Model

In this section, we first give a model of the LEO satellite constellation and its characteristics, based on which research scenario is depicted. After that, we describe the traditional Q learning algorithm and its application in routing problems. The definition of the routing problem is given in the last sub-section.

### 2.1. LEO Satellite Constellation

Currently, LEO satellite constellations can be divided into two types based on the number of satellite orbital planes: single-layer and multi-layer constellations. The Iridium system is representative of single-layer constellations, while Starlink is a multi-layer constellation with satellite orbital planes mainly distributed between 300 and 600 km. To facilitate the analysis, this paper uses the Iridium system as the satellite environment.

**Figure 1:**
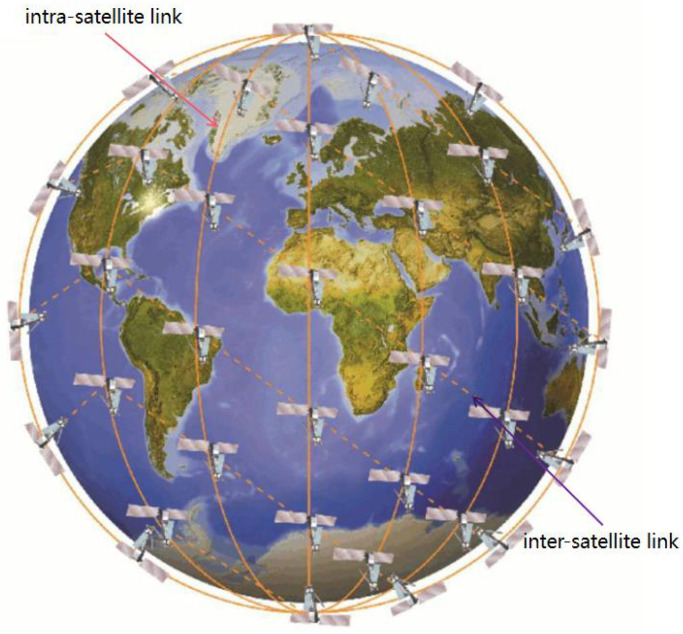
LEO satellite constellation.

As shown in Figure 1, the Iridium constellation consists of multiple satellite orbits that are evenly distributed which intersect at the pole position. The area at the ends of the constellation is known as the polar region, where satellites are prohibited from communicating. The direction of motion of the satellite changes as it passes the pole, and the relative positions between the satellites change, which leads to periodic changes in the topology of the satellite. There are two satellite-to-satellite links within the satellite network. The link with an orbiting satellite is an intra-satellite link, and the link between adjacent orbiting satellites is an inter-satellite link [20]. Thus, each satellite has up to four neighbor nodes with which to communicate, leaving little decision space for the satellite when considering packet forwarding.

### 2.2. Q-routing Algorithm

Reinforcement learning is one of the areas in machine learning [21]. Inspired by behavioral psychology, it focuses on what actions an agent should perform to maximize cumulative rewards when confronted with a given environment. Reinforcement learning consists of agent, environment, state, action, and reward, where the environment consists of some states. The agent performs an action based on the current state of the environment, after which the environment moves to the next state based on the action performed and provides the agent with a reward value to evaluate the action. In the long run, the agent learns how to perform the optimal action in a given state [22].

Q learning is a traditional reinforcement learning algorithm; it provides the method by which the intelligence chooses actions in a given state by maintaining a Q-table. Each Q value in the Q-table represents the total benefit of taking a certain action in a certain state. The update of the Q value is mainly realized through the Bellman Equation:

(1) $$Q\left(s,a\right)=\left(1-\alpha \right)Q\left(s,a\right)+\alpha [r+\gamma \max_{d\in A}Q\left(s^{{}^{\prime }},a^{{}^{\prime }}\right)]$$

where *s* represents the current state of the agent, *a* is the action performed, α is the learning rate, *r* is the reward value by performing action *a* under state *s*, γ represents the discount factor, and *A* is the action space in state $s^{{}^{\prime }}$; α and γ both are in the interval [0, 1]. Therefore, max $Q(s^{{}^{\prime }},a^{{}^{\prime }})$ represents the max *Q* value of state $s^{{}^{\prime }}$.

Q routing is the application of the Q learning algorithm to the routing problem. In the Q routing algorithm, each communication node is treated as an agent which can independently learn the forwarding policy and forward packets to the next port [23]. As shown in Table 1, each node maintains a Q-table, which records the Q value of all actions and states. The agent looks up the Q-table based on the destination node of the packet, finds the action corresponding to the maximum Q value, and then executes it, which is a packet forwarding process. Similar to Equation (1), the update of Q-table is as follows:

(2) $$Q_{i}\left(s,a\right)=\left(1-\alpha \right)Q_{i}\left(s,a\right)+\alpha \left[r+\gamma \max_{d\in A}Q_{j}\left(s^{{}^{\prime }},a^{{}^{\prime }}\right)\right]$$

where α is the learning rate which determines the updating rate, γ is discount factor, and *i*, *j* represent the index of different nodes. This equation is the essence of agent learning.

### 2.3. Problem Statement

The LEO satellite network can be represented as graph G = (V,E), where V represents the set of satellite nodes and E represents the set of satellite links. Consider an Iridium-like system with M number of orbits and N satellites per orbit; we use (*i*, *j*) to represent the position of a satellite, where i represents the satellite’s orbit number and *j* represents the satellite’s number in orbit (1≤i≤M,1≤j≤N). There are intra-satellite links between satellites in the same orbit and inter-satellite links between satellites in different orbits, which means that each satellite can communicate directly with up to four satellites. For clarity, we list the notations and definitions in Table 2.

In this article, we only consider the process of packet transmission between satellites. The packet starts from the initial node $N_{i}$; the node first finds the next hop node $N_{j}$ from the set of neighbor nodes $N_{gi}$, and then sends the packet out. The transmission delay of the packet is $D_{ij}$, and then the next hop node repeats the previous action, sending the packet to its neighbor node, and then updating the transmission delay of the packet D according to the delay accumulation rule. The above steps are repeated until the data packet is forwarded to the destination node. The problem is planning a route which minimizes D. In a real-world scenario, thousands of packets are passed through the network, so the algorithm needs to consider the congestion of the link. Firstly, the algorithm must be able to plan a feasible path from the source node to the destination node, and secondly, the algorithm should minimize the delay of this path, including propagation delay and queuing delay. Therefore, the ultimate goal of the algorithm is to minimize the transmission delay of packets while ensuring the packet arrival rate.

## 3. Proposed FRL–SR Algorithm

In this section, we first discuss the design of the reinforcement learning model, including states, reward functions, and actions in Section 3.1. Then, we briefly introduce the neighborhood discovery and explain the propagation range of ‘hello’ packets in reinforcement learning in Section 3.2. The training approach and algorithm are proposed in Section 3.3 and Section 3.4. In Section 3.5, we analyze the time complexity and space complexity of the FRL–SR algorithm and the Dijkstra algorithm.

### 3.1. Reinforcement Learning Model Setting

In solving the satellite routing problem using multi-agent reinforcement learning, we consider each satellite node as an agent. When the data packet arrives at the satellite node, it observes the current state and forwards the packet based on the present situation. The node adjusts the forwarding strategy according to the current state of the network. Once a link is broken or there is congestion present, the reward of this port is decreased, and packets are forwarded to another path. This is the main advantage of reinforcement learning compared with traditional routing algorithms.

The entire packet forwarding process can be viewed as a finite-state Markov decision process (MDP) whose final state occurs when the packets have arrived at the destination node. We use (S, A, P, R) to represent a state of the MDP, where S is the state of the current system, A is the action space, P is the probability of state transition, and R is the reward. Each satellite node only forwards packets to its neighbor nodes, which means that the action space is up to four. Therefore, we chose reinforcement learning rather than deep reinforcement learning to achieve this.

When using reinforcement learning to solve problems, it is crucial to design state, action, and reward functions [24]. For different scenarios, we should follow different design principles. In our satellite network routing scenario, the states, actions, and rewards are defined as follows:

- States: Each state $s_{t}\in S=\left\{N_{c},N_{d},q_{1}^{t},q_{2}^{t},\ldots ,q_{P}^{t}\right\}$ indicates the present situation in the satellite network environment, where $N_{c},N_{d}$ represent current node and destination node for packet, respectively. The parameter $q_{p}^{t}$ represents the current queue length of the *p*-th node for *p* = 1 to *p* = *P*. In multi-agent reinforcement learning, each agent observes a different state, and they make independent routing decisions based on the current state;
- Actions: The action $a_{t}\in A=\left\{p_{1},p_{2},\ldots ,p_{P}\right\}$ represents the agent choosing a node from its neighborhood nodes for each upcoming packet, where $p_{p}$ represents the p-th satellite node. In satellite networks, each satellite has up to four neighbor nodes, so the length of A is up to four [25];
- Reward: The reward function is designed according to the goal we want to achieve, such as the minimum time delay and the maximum packet arrival rate. In this paper, the transmission delay consists of two parts, propagation time and waiting time, so the reward function consists of the following three parts:
  – Propagation delay. In satellite networks, the star link often fails and becomes unavailable, which requires frequent inter-satellite reconnections. For convenience, we consider both reconnection time and propagation time as propagation time delay. $D_{ij}$ represents propagation delay between node $N_{i}$ and node $N_{j}$;
  – Queue length. Each satellite node maintains the receiving queue $q_{r}$ and the transmitting queue $q_{t}$. Queuing delay occurs when the number of packets received by the satellite is larger than the length of the satellite receiving queue. The agent learns to forward data packets to satellite nodes with small queue length to reduce the waiting time;
  – Load growth. To avoid packets being forwarded centrally to individual satellite nodes, causing network congestion, we record the receiving queue length in the previous stage as the load growth of the satellite, which is recorded as $g_{i}$. Therefore, $g_{i}$ could be seen as the congestion level of nodes $N_{i}$. This avoids the situation that everyone sends data to “high-quality” nodes at the same time.

Equation (3) gives the expression of reward function, where $q_{max}$ represents the maximum queue length, and $w_{1}$ and $w_{2}$ represent the growth and delay coefficients, respectively. When the next hop is the destination node, we set the reward to 20*N*, which ensures that the packets are transmitted to the destination node as soon as possible.

(3) $$reward_{j}=\left\{\begin{array}{cc} 20N & N_{j}\;\mathrm{is}\;\mathrm{the}\;\mathrm{destination}\\ q_{max}-\left(q_{r}+q_{t}\right)-w_{1}*g_{j}-w_{2}*D_{j} & \mathrm{Otherwise} \end{array}\right.$$

**Figure 2:**
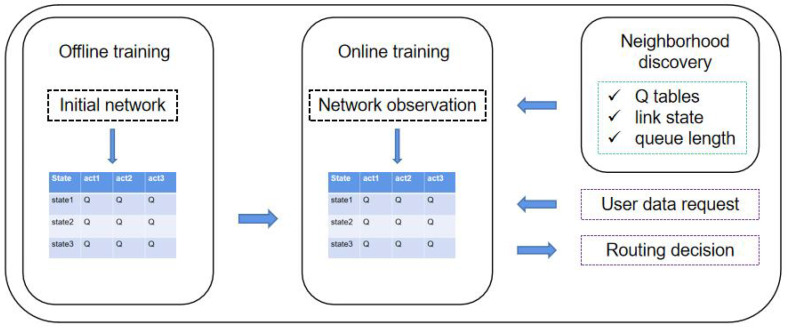
Model framework of FRL–SR.

As shown in Figure 2, the system framework consists of three parts: Neighbor Node Discovery, Offline Training, and Online Training. In multi-agent reinforcement learning, the learning process of the agent needs the information of the neighboring agents, which includes Q-table, connection status, and available resources. Therefore, we develop the neighborhood discovery part for agents to share information. During the offline training phase, we perform the initial training of the agents in a ground-based network environment. By randomly generating packets, the agents act by observing the state of the packets and the state of the neighboring nodes. To avoid local optimal solutions, we use the ϵ-greedy strategy to explore a larger unknown space. Static network training results are not fully suitable for dynamic LEO satellite networks, so online training is required to fine-tune the Q-table. Agents make routing decisions for satellite networks based on the pre-trained model and update the Q-table with the feedback results. It is important to note that the ϵ-greedy strategy is not used at this stage, as we only need to fine-tune the Q-table. The advantage of using the pre-trained method is that it saves onboard resources and improves the performance of the initial phase of the algorithm.

### 3.2. Neighborhood Discovery

Since the topology of the satellite network changes dynamically and the links between satellites are unstable, the neighbor nodes of the satellite change. Therefore, agents must periodically check the neighbor nodes so that they know where to forward packets when they arrive.

Satellite nodes receive information about their neighbors by receiving ‘hello’ packets from neighboring nodes [26]. In addition, the “hello” packet contains a Q-table, link information, and available resources that nodes can use to calculate reward values and update their own Q-table. If a node does not receive “hello” packets from a neighboring node for a long time, it assumes that the neighboring node is down and will not send packets to it later. In this paper, nodes only send ‘hello’ packets to their neighbors. Compared to flooding ‘hello’ packets in terrestrial networks, this method saves energy cost for the satellite and does not burden the network.

### 3.3. Offline Training

The algorithm proposed in this paper needs offline training before being applied to satellite nodes. The purpose of this process is to reduce the training time of the algorithm online and improve its initial performance. The network $G_{t0}$ at $t_{0}$ time is input as the initial state, and the output is that each satellite node receives a Q-table.

To simulate the random nature of packet generation, the initial and destination nodes of packets are randomly selected from the set of satellite nodes. To reduce training time, if a packet is forwarded to its destination node, a new packet is generated, and its initial node and destination node are also randomly generated.

To better explore the unknown space, the ϵ-greedy strategy is used in the offline training phase. As shown in Equation (4), the agent randomly chooses an action with probability ϵ, and, with probability 1−ϵ, it chooses the action with the maximum q-value. This strategy prevents local optimal solutions, but the convergence speed is low. To solve the above problem, the value of ϵ gradually decreases as reinforcement learning progresses, which can speed up the convergence of the algorithm without affecting the learning effect.

(4) $$a_{t}=\left\{\begin{array}{cc} \mathrm{random}\;\mathrm{action} & w.p.\epsilon \\ argmax_{a}Q_{t+1} & w.p.1-\epsilon \end{array}\right.$$

Algorithm 1 is the process of offline training. First we need to initialize the training parameters: $num_{step}$ is the total number of steps trained, ϵ is the probability value of the greedy strategy, $l_{r}$ is the learning rate of reinforcement learning, and γ is the discount factor of the Q value update function. At each step, the algorithm first cleans up the remaining packets in the network and then randomly generates a preset number of packets. For each satellite node, we first determine whether its sending queue $q_{t}$ is empty, and the top packet pops up when it is not empty. Then, we select the next hop node m according to the ϵ-greedy strategy. If the receive queue $q_{r}$ of node m is not full, the packet is forwarded to m, and the current node will receive the Q-table and reward value of node m. After that, the Q-table of the current node should be updated according to Equation (2). Otherwise, the network is congested and packets will be inactive until the next round arrives. 
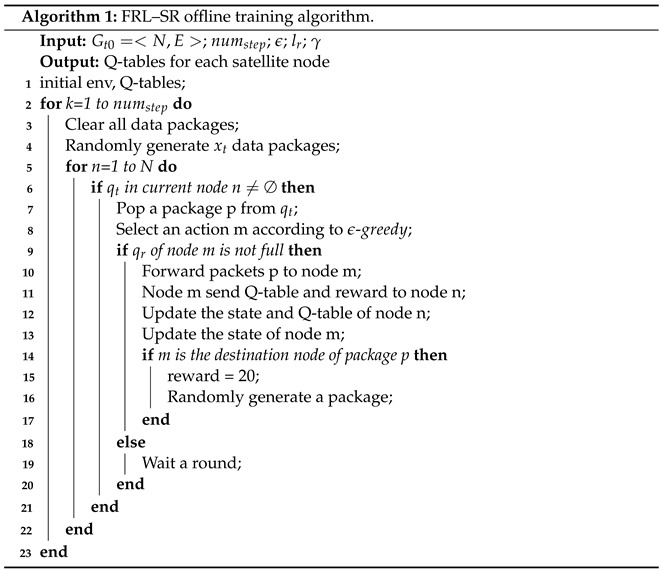


### 3.4. Online Training

In the online training phase, the pre-trained parameter in Section 3.3 is used as the initial routing decision strategy. The online training algorithm carries out two things: one is to make routing decisions based on the pre-trained model, and the other is to fine-tune the Q-table based on the real satellite network environment.

Unlike offline training, agents in online training do not make decisions according to the ϵ-greedy strategy, since agents avoided the local optimal solution in the previous step. The main purpose of this process is to fine-tune the routing strategy. Moreover, the agents are already making routing decisions at this stage, and the algorithm must ensure that these decisions are sound.

We simplify the satellite network routing problem to finding the smallest delay path. The delay of a packet consists of two parts: propagation delay and queuing delay. The propagation delay depends on the inter-satellite link distance and link status, and the queuing delay depends on the available resources. According to Equation (3), we know that the reward value is linearly related to the delay of the packet. Equation (5) is the definition of the Q value in reinforcement learning, from which we can conclude that the Q value represents the estimated total return of an action in the current state, and the larger the Q value, the smaller the delay. Therefore, the path with the maximum Q is the path with the minimum delay. According to the greedy algorithm, if each agent chooses the action with the largest Q value, the latency of the obtained packet is close to the optimal choice.

As shown in Equation (6), we use $Q_{sum}$ to represent the goal of algorithm optimization, which is a linear combination of the Q values of each agent. Combined with Equation (5), we can derive the relationship between $Q_{sum}$ and each reward value. If we know that the reward value and delay are linear, then $Q_{sum}$ and delay are also linear, so we only need to maximize $Q_{sum}$ to obtain the minimum delay path.

(5) $$Q_{i}\left(s,a\right)=R_{i}+\gamma \left(R_{i+1}+R_{i+2}+\ldots +R_{n}\right)$$

(6) $$Q_{sum}=\sum_{i=1}^{n}w_{i}Q_{i}\left(s_{i},a\right)$$

If we suppose that the link state of a satellite changes, it is obtained first by the two satellite nodes of this link, followed by the neighboring nodes of the two satellite nodes. Therefore, the state of links is serial propagation, which causes certain difficulties for the convergence of the reinforcement learning algorithm. Especially, the state of the satellite network is prone to change; it is possible that the convergence of the previous stage is not yet complete and the link state has changed again.

**Figure 3:**
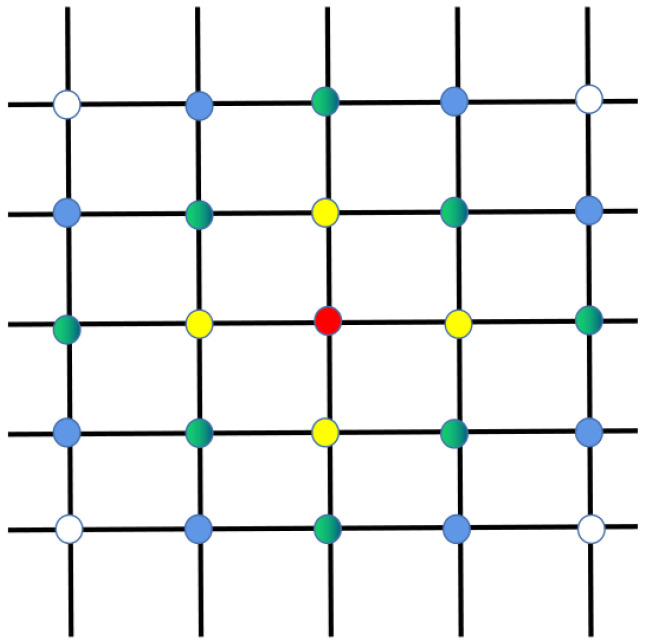
Schematic diagram of link state propagation.

As shown in Figure 3, there are five orbits, each with five satellites. Each satellite is represented by a dot with a different color. The red node satellite transmits a message outwards, and the first to receive that message are the yellow nodes around it. The yellow node then passes the message to the green node, and the white node does not receive the message until the fourth round of message dissemination. This indicates that the message transmission in the satellite network is linear. In the traditional Q-routing algorithm, the agent receives the Q-table and link state information feedback from the neighbor nodes when and only when it sends a packet to its neighbor nodes. Then, the node updates its Q-table, which is a learning process. This paper proposes a method named empty packet convergence method to accelerate the perception of the network state by agents. Neighboring nodes do not only send status information after receiving a packet, but also broadcast its message by period t. The traditional learning process only updates a certain item of the two-dimensional Q-table at a time, while the empty packet convergence method updates the entire content of a node’s action space at a time. The smaller the t, the more often agents perceive the network. Therefore, we designed t to be inversely proportional to the node traffic density; the higher the traffic density, the smaller the t, and the faster agents perceive the network. This ensures faster awareness of the state of the network without increasing the network overhead too much.

Algorithm 2 is the pseudocode of the online learning process of the algorithm. It differs from the traditional reinforcement learning algorithm in that this paper proposes an empty packet convergence method, which accelerates the obtaining of network status and is more suitable for the state-variant satellite network environment. 
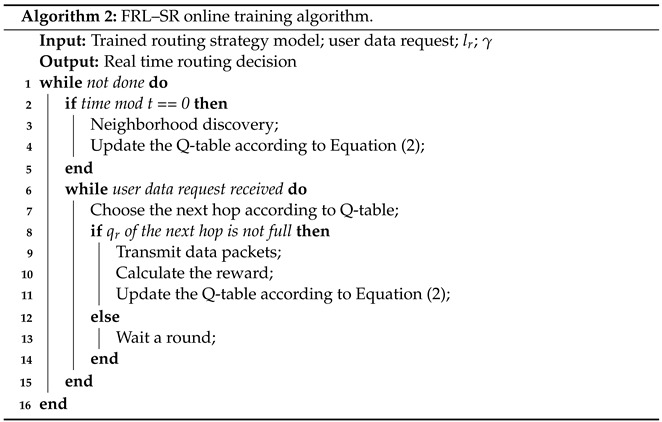


### 3.5. Complexity Analysis

In this paper, we compare FRL–SR with the Dijkstra algorithm in terms of algorithm complexity. The time complexity of the Dijkstra algorithm is $O(n^{2})$: it has to obtain global state information and then compute the shortest path from the current node to any node through two layers of loops. The FRL–SR algorithm proposed in this paper only queries the Q-table stored in the satellite when making routing decisions, so the time complexity is constant. Therefore, FRL–SR is more suitable for solving routing problems for large satellite constellations. In terms of spatial complexity, both of them store a two-dimensional array. The spatial complexity of the Dijkstra algorithm is O(E +4N) when using adjacency tables for data storage, where E is the number of edges in the network diagram. The maximum action space of the FRL–SR algorithm is 4, so the space complexity is O(4N), which does not take up more storage space than the Dijkstra algorithm.

Another important indicator is the communication overhead during the execution of the algorithm. In the Dijkstra algorithm, each satellite node must send its own status information in a flood so that each satellite node can receive the global information. The communication overhead is high and also has some negative impact on the network load. In the FRL–SR algorithm, each satellite node only needs to send the status information to its neighboring nodes without forwarding it to the whole network. Even if we increase the number of communications between neighbor nodes to improve the convergence speed, it is much lower than the communication overhead of the Dijkstra algorithm.

## 4. Simulation Results

In this section, we present the simulation results of the FRL–SR algorithm. The experiment was mainly divided into two parts: First, we simulated different reward functions in reinforcement learning and found out which one could minimize the delay for our subsequent experiments. Then, we compared the performance differences between the FRL–SR algorithm proposed in this paper and the traditional Dijkstra algorithm, both running in the same network environment and with the same user data requirements. We compared these two algorithms in terms of average delay, packet arriving ratio, and network load balance, and explained the reasons for their performance differences. To make the experimental results more accurate, we repeated all the experiments three times and took the average of the results as the final result.

In this paper, we chose the Dijkstra algorithm as the comparison algorithm because it is the most mature and widely used algorithm in satellite routing algorithm, and through experiments, we verified that the proposed algorithm can effectively improve the satellite routing strategy and better support communication services [27].

The parameters of the experiment are given in Table 3. The network had a total of 7 satellite orbits, each containing 7 satellites, for a total of 49 satellites [28]. Because interstellar links fail frequently and link recovery varies, to simulate a satellite network more realistically, we set the propagation delay to vary according to a sinusoidal curve. In the offline training phase, the algorithm ran for 30 episodes—the step for each episode was 200, which ensured each agent could learn the routing strategy in a limited number of training steps. In the online training phase, we observed the delivery of packets in the network every 1 s and recorded it. In order to ensure the stability of the network, we adjusted the learning rate of this stage to 0.2, and the corresponding learning rate of the offline training stage was 0.8.

**Figure 4:**
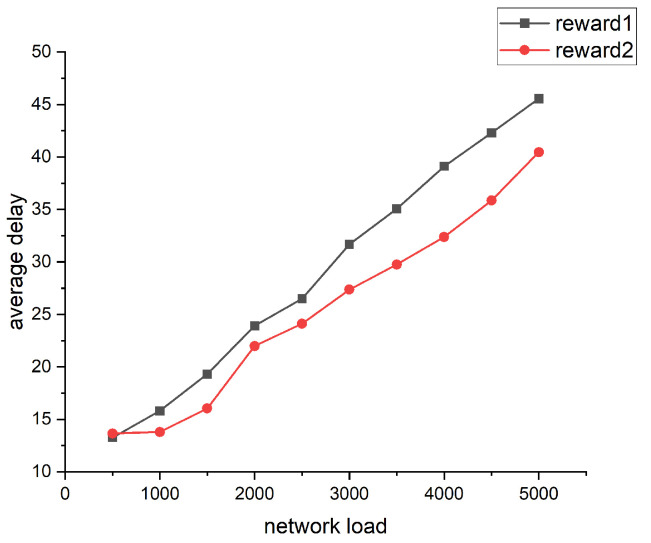
Comparison of different reward performances.

The performance of different rewards is shown in Figure 4. The reward1 function was designed to be related only to the length of the link between the two nodes, in which $D_{ij}$ was the distance between node *i* and node *j*.

(7) $$reward1=\left\{\begin{array}{cc} 980 & \mathrm{Next}\;\mathrm{node}\;\mathrm{is}\;\mathrm{the}\;\mathrm{destination}\\ -0.1*D_{ij} & \mathrm{Otherwise} \end{array}\right.$$

The *reward2* function was inversely relative to the queue length of the node and the distance between the two nodes.

(8) $$reward2=\left\{\begin{array}{cc} 980 & \mathrm{Next}\;\mathrm{node}\;\mathrm{is}\;\mathrm{the}\;\mathrm{destination}\\ 300-\left(q_{r}+q_{t}\right)-5*g_{j}-0.1*D_{ij} & \mathrm{Otherwise} \end{array}\right.$$

Based on the simulation results, we can conclude that the design of the reward function has a great influence on the performance of the algorithm. The goal of the algorithm was to obtain the path with the least delay, so we chose the second reward function for subsequent simulations.

**Figure 5:**
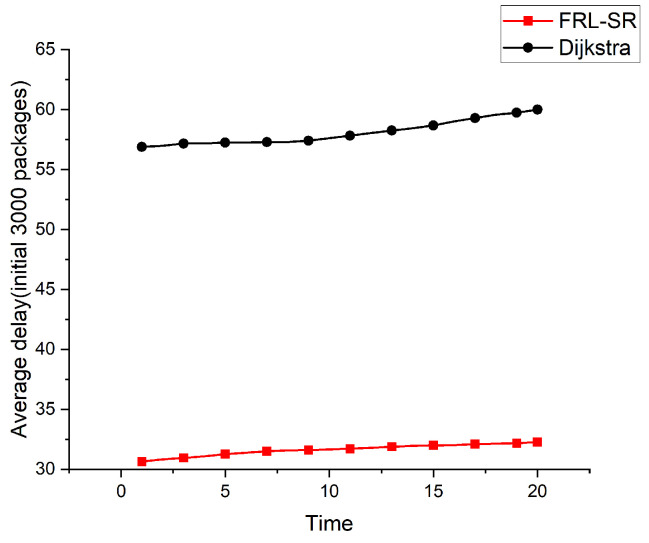
Comparison of the average delay of the FRL–SR algorithm and the Dijkstra algorithm with 3000 initial packages.

**Figure 6:**
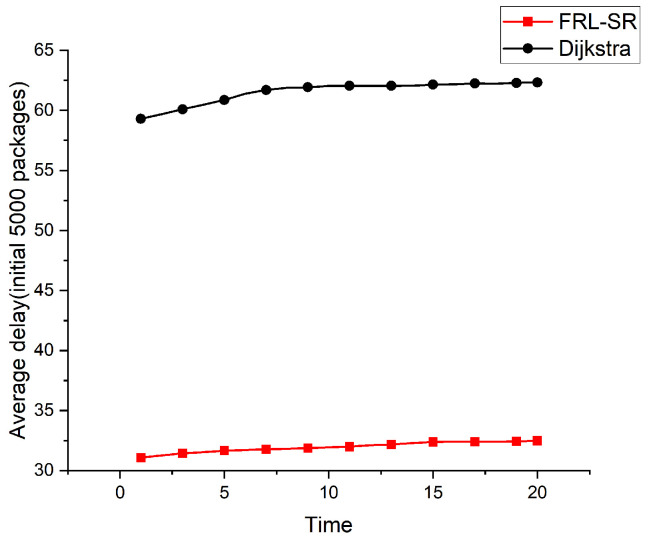
Comparison of the average delay of the FRL–SR algorithm and the Dijkstra algorithm with 5000 initial packages.

Both Figure 5 and Figure 6 show the relationship between the average delay and time in operation for the FRL–SR algorithm and the Dijkstra algorithm. To better demonstrate the actual effect of the algorithm, we took the study time after the network was relatively stable, rather than when the system was first put into operation. The initial number of packages in Figure 5 is 3000, and the initial number of packages in Figure 6 is 5000. We can see that the FRL–SR algorithm showed consistent performance in environments with different initial packet counts.

The FRL–SR algorithm outperforms the Dijkstra algorithm in terms of average delay in the same network environment. This is because the FRL–SR algorithm adjusts its forwarding policy based on the current state of the network and then forwards the packets to the optimal port, while the Dijkstra algorithm makes forwarding decisions based on the previously collected global information. The link state of the satellite network changes rapidly, and the information collected by Dijkstra cannot accurately reflect the state of the satellite network at that time. As can be observed from the above graph, the average latency increases slowly with time because the inter-star link is prone to failure, resulting in packet loss. The algorithm in this paper does not have a data retransmission function, which means the delay of lost packets will keep increasing, resulting in a rising average delay.

**Figure 7:**
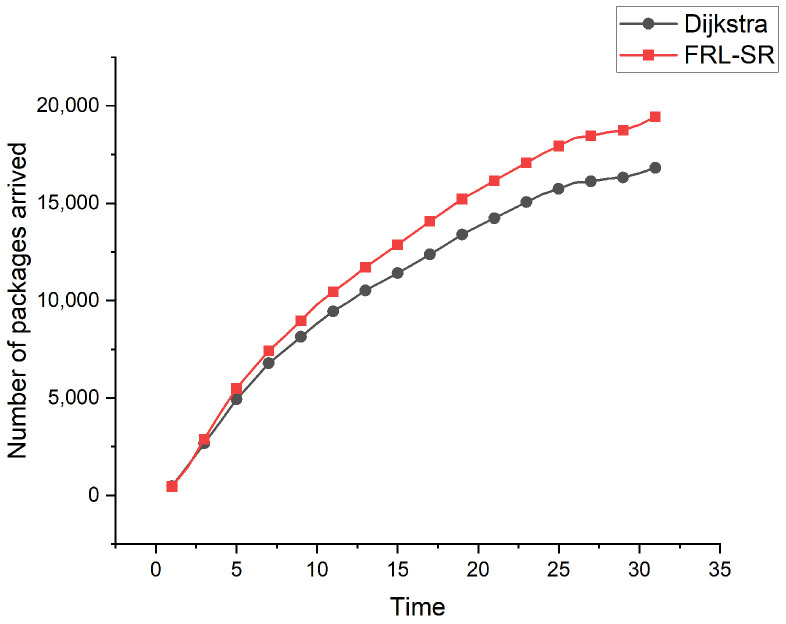
Comparison of the number of successfully delivered packets by FRL–SR algorithm and Dijkstra algorithm.

The relationship between cumulative number of packets and time is shown in Figure 7. We can see that, over time, the advantages of the FRL–SR algorithm over the Dijkstra algorithm gradually become apparent. Each satellite node has limited resources, so the communication capacity of the whole network is also limited. The FRL–SR algorithm considers the resource utilization of satellite nodes as an optimization objective. When a satellite node selects the next hop, it takes the queue length of neighboring nodes as a consideration parameter and forwards the packets to the satellite with sufficient communication resources first, which improves the resource utilization of the network and forwards more packets per unit time.

Considering Figure 5, Figure 6 and Figure 7 together, we observe that the FRL–SR algorithm transmits a higher number of successful packets in the same amount of time with a smaller average delay per packet. This is enough to show the advantages of the FRL–SR algorithm proposed in this paper over the traditional satellite routing algorithm.

(9) $$s=\sqrt{\frac{\sum {\left(x-M\right)}^{2}}{n}}$$

**Figure 8:**
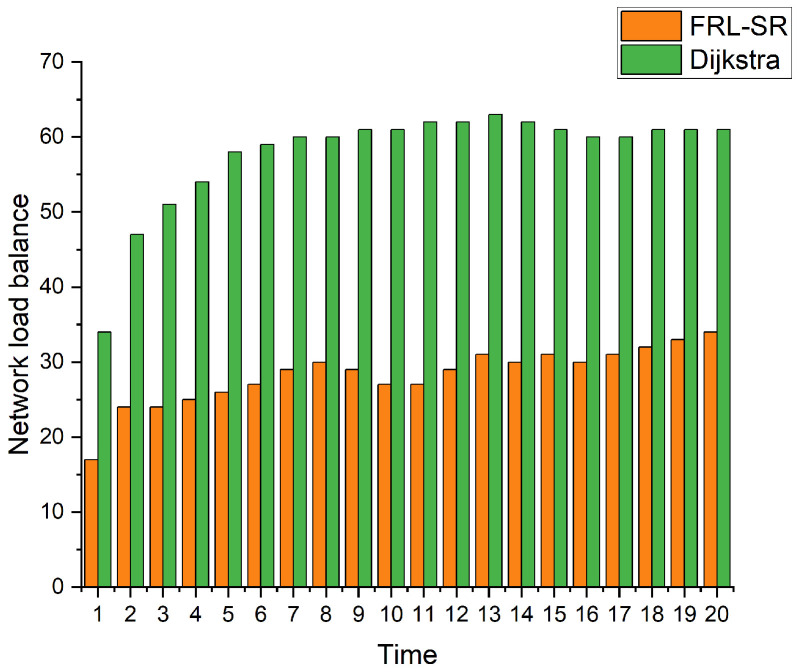
Measure the balance of node load in the network.

Figure 8 shows a comparison of the load balance of nodes in the network. We use the population standard deviation to express the load balance of the network, as shown in Equation (9), where M is the mean of the data and n is the total number of nodes in the network, which is a commonly used parameter to describe the degree of discreteness of the system. By observing the simulation results, we can observe that, under the same user request conditions, the FRL–SR algorithm has a better load balancing effect than the Dijkstra algorithm. The former can make full use of the resources of each node for routing, while the Dijkstra algorithm is more likely to cause network congestion.

Based on the above simulation results, we conclude that the FRL–SR algorithm has lower network latency and higher data throughput, which is more suitable for satellite networks with variable network status. In addition, the FRL–SR algorithm also has good load balancing characteristics, and it considers both satellite link status and satellite load when making data forwarding decisions, avoiding network congestion. The Dijkstra algorithm only blindly forwards packets to ports with good link status, causing congestion on some network links.

## 5. Conclusions

In this paper, we proposed a fast-convergence reinforcement learning algorithm to construct the routing issue in the LEO constellation. Aiming at addressing the characteristics of large dynamic satellite network status and unstable inter-satellite links, we designed a routing method named the fast-convergence reinforcement learning satellite routing (FRL–SR) for online decision-making. This method is always aware of the network link status and dynamically adjusts its routing strategy. The FRL–SR algorithm includes three parts: neighbor node discovery, offline training, and online training. By shortening the cycle time of agent obtaining network states, we accelerated the convergence speed of the algorithm, so that the routing decision was more suitable for the current network state. In addition, we also performed a complexity analysis, and the FRL–SR algorithm was superior to the Dijkstra algorithm in both time complexity and spatial complexity.

The simulation results showed that the FRL–SR algorithm had a lower average delay and higher packet arriving ratio compared with the Dijkstra algorithm. In addition, the FRL–SR algorithm also had a good performance with respect to load balancing. It made full use of the resources of each node and reduced the probability of network congestion. Multi-agent cooperation is a promising method to solve the problem of large-scale satellite network routing. In future work, we will continue to work on multi-agent reinforcement learning algorithms to better solve the problem of satellite network routing.

## Figures and Tables

**Table 1:** Q-table of node *i*.

State	Neighbor			
	$N_{1}$	$N_{2}$	$N_{3}$	**…**
($i,D_{1}$)	$Q_{i}\left(\left(i,D_{1}\right),N_{1}\right)$	$Q_{i}\left(\left(i,D_{1}\right),N_{2}\right)$	$Q_{i}\left(\left(i,D_{1}\right),N_{3}\right)$	…
($i,D_{2}$)	$Q_{i}\left(\left(i,D_{2}\right),N_{1}\right)$	$Q_{i}\left(\left(i,D_{2}\right),N_{2}\right)$	$Q_{i}\left(\left(i,D_{2}\right),N_{3}\right)$	…
($i,D_{3}$)	$Q_{i}\left(\left(i,D_{3}\right),N_{1}\right)$	$Q_{i}\left(\left(i,D_{3}\right),N_{2}\right)$	$Q_{i}\left(\left(i,D_{3}\right),N_{3}\right)$	…
(i,…)	…	…	…	…

**Table 2:** Notations of variables.

Notations	Definition
G	graph of the LEO satellite network
V	set of satellite nodes
E	set of satellite links
M	number of orbits
N	number of satellites per orbit
$D_{ij}$	transmission delay between node *i* and node *j*
$Ng_{i}$	the set of neighbors of node *i*

**Table 3:** Simulation parameters.

Parameters	Values
Number of satellites	49
Delay type	sinusoidal
Trials	3
Offline training network load	3000
Initial network load for online training	3000
Max queue length	150
Max transmit packages at one time	10
Number of episodes	30
Number of steps peer episode	200
discount factor	0.9
Learning rate for offline training	0.8
Learning rate for online training	0.2

## Data Availability

The relevant data of this paper can be contacted by dingzhl@shanghaitech.edu.cn.
